# 848 Polylactic Acid Skin Substitutes for Pediatric Burns: A Retrospective Review and Meta-Analysis

**DOI:** 10.1093/jbcr/iraf019.379

**Published:** 2025-04-01

**Authors:** Anh-Tho Antoinette Nguyen, Derek Bell

**Affiliations:** Kessler Burn Center; Kessler Burn Center

## Abstract

**Introduction:**

Pediatric burn injuries are a significant source of morbidity, with advanced wound care materials such as polylactic acid alloplastic skin substitutes showing promise in accelerating healing, reducing infection rates, and improving patient outcomes. This study integrates a retrospective chart review of pediatric burn patients treated with a polylactic acid membrane with a systematic review and meta-analysis of studies examining the efficacy of polylactic acid alloplastic skin substitutes in pediatric burn care.

**Methods:**

A retrospective chart review was conducted at an ABA-verified pediatric burn center from January 2022 to January 2024, encompassing 47 pediatric patients. Descriptive analyses assessed patient demographics, burn characteristics, healing times, infection rates, and hypertrophic scarring. The systematic review incorporated 10 studies from Embase, Scopus, and PubMed, with 463 pediatric patients included in the meta-analysis. Pooled outcomes assessed infection rates, healing times, pain scores (VAS), and hypertrophic scarring. Random-effects models were applied to account for between-study heterogeneity, and I² was used to assess variability.

**Results:**

The mean age of this cohort was 5.9 ± 3.2 years and 61.7% were male. Patients treated with polylactic acid skin substitutes exhibited a mean healing time of 10.2 ± 3.1 days. The infection rate was 6.4%, and hypertrophic scarring occurred in 11% of the patients. The mean inpatient length of stay was 6.8 ± 4.2 days, with a postoperative stay of 0.44 ± 0.14 days. The meta-analysis revealed a pooled weighted mean healing time of 14.0 days (95% CI: 12.5-15.5; I² = 55%, p = 0.05) for polylactic acid alloplastic skin substitutes, consistent with findings from the retrospective study. Pain scores showed a pooled weighted mean difference (WMD) of 2.0 (95% CI: 1.4-2.6; I² = 45%). The pooled infection rate was 7% (95% CI: 5%-11%; I² = 59%), comparable to the infection rate in the retrospective cohort. Hypertrophic scarring was observed in 12% of patients treated with polylactic acid alloplastic skin substitutes (95% CI: 8%-17%; I² = 0%).

**Conclusions:**

The retrospective chart review and meta-analysis demonstrate that polylactic acid alloplastic skin substitutes significantly improve pediatric burn care by reducing healing times, infection rates, and hypertrophic scarring. These materials enhance recovery outcomes, decrease hospital stays, and offer cost-effective care. With their ability to promote rapid healing and minimize complications, polylactic acid substitutes hold promise as an essential component in pediatric burn treatment. Further large-scale studies are warranted to confirm these benefits and explore long-term outcomes.

**Applicability of Research to Practice:**

This research supports using polylactic acid skin substitutes in pediatric burn care to improve healing, reduce infections, and shorten hospital stays, enhancing patient outcomes and efficiency in clinical practice.

**Funding for the Study:**

N/A

